# Analysis of Parents’ Attitudes and Knowledge toward Immunization and How These Factors Influence Their Decisions to Vaccinate Their Children against Human Papilloma Virus (HPV)

**DOI:** 10.3390/medicina59101755

**Published:** 2023-09-30

**Authors:** Beata Zastawna, Anna Milewska, Roman Załuska, Remigiusz Kozłowski, Martyna Zastawna, Michał Marczak

**Affiliations:** 1 Department of Management and Logistics in Health Care, Medical University of Lodz, 90-131 Lodz, Poland; roman.zaluska@stud.umed.lodz.pl (R.Z.); remigiusz.kozlowski@umed.lodz.pl (R.K.); 2 Department of Statistics and Medical Informatics, Medical University of Bialystok, 15-089 Bialystok, Poland; anna.milewska@umb.edu.pl; 3 Department of Psychology, SWPS University of Social Sciences and Humanities, 03-815 Warsaw, Poland; mzastawna@st.swps.edu.pl; 4 Collegium of Management, WSB University in Warsaw, 03-204 Warsaw, Poland

**Keywords:** hesitancy, vaccination, HPV, acceptance of vaccination, antivaccine myths

## Abstract

*Background and objectives*: Vaccination hesitancy is a growing problem associated with decreasing chances for the elimination of vaccine-preventable diseases through immunization. This research was conducted to learn about parents’ attitudes toward vaccination, particularly against HPV, in the context of their beliefs and fears regarding immunization and the impact of these views on their children’s vaccination decisions. An additional goal was to determine what views or convictions most often lead to the phenomenon of delaying or refusing vaccinations, especially HPV vaccines, in the surveyed community. *Materials and Methods*: An online questionnaire was conducted in which parents marked how they vaccinated their children with mandatory vaccinations and whether they took the opportunity to protect their children with additional vaccinations, including HPV and COVID-19. Respondents also marked the extent to which they agreed with the statements, regarding the safety of vaccinations, doubts about them, as well as popular anti-vaccine myths. *Results*: A total of 250 questionnaires were collected with data on the immunization of 425 children. Associations between specific beliefs and vaccine abstention were established. The most motivating factor for vaccination against HPV was the parent’s awareness that the virus HP is the cause of cancer. The most inhibiting factor was respondent’s compliance with the opinion that HPV vaccines can affect the child’s sexual activity. *Conclusions*: The results indicate that information campaigns are needed, especially focusing on vaccine safety and the regularity and transparency of monitoring adverse effects. The obtained results will be used to create educational interventions against vaccination hesitancy more adapted to local conditions.

## 1. Introduction

The health programs of the World Health Organization (WHO) and all cooperating countries include improving the health and increasing the life expectancy of people by, among other things, eliminating the most common and most dangerous infectious diseases. The most effective and safe method to achieve this is immunization, which confers protection upon the vaccinated person and, on the other hand, reduces the risk of the transmission of pathogens throughout the community. An adequate vaccination rate of the population also protects non-immune individuals from the threat of infection—the phenomenon known as “herd immunity” [1,2].

The confirmation of the role of some chronic viral infections in the processes of oncogenesis has given hope that, through immunization, there will also be the possibility of reducing cancer [3,4,5,6,7]. The introduction of widespread hepatitis B vaccination has reduced the number of infections, including chronic viral hepatitis B and consequently the incidence of hepatitis B-dependent primary liver cancer, especially in endemic countries [6,8,9,10]. Another virus, the role of which in carcinogenesis has been confirmed, is the human papilloma virus (HPV), specifically the individual highly oncogenic serotypes responsible for cervical cancer in women and cancers of the urogenital area and in the head and neck localization in both sexes [11,12,13,14,15,16]. Additional risk factors, including smoking, even second-hand smoke [17], and the disorders in the microbiota of the urogenital tract or/and in lower intestinal section [18], reduce local immunity. This can promote the transition of the HPV infection into persistent form and increase the possibility of oncogenesis.

In EU/EEA countries, 3300 cases of cervical cancer and more than 13,000 deaths from it are diagnosed annually. In Poland, cervical cancer is a common neoplasm, with about 3500 new cases diagnosed annually (in 2020–3862 cases), and it is the second most common neoplasm in women under the age of 50. Despite existing prophylactic cytological screening programs, which are attended regularly only by 18% of Polish women, the disease is usually detected in later stages, so the mortality rate of patients in Poland is high: 2137 deaths were reported in 2020 [19,20].

With the creation of the HPV vaccine in 2006, there was an opportunity to minimize the risk of cervical cancer in women, as well as other cancers caused by these viruses. Recent reports suggest that HPV infection may also be one cause of infertility [21,22]. Ongoing studies of the impact of HPV vaccination on people with active lower genital tract infections have shown that vaccines can have not only a preventive but also a therapeutic effect [23,24].

According to the plan included in the National Oncology Strategy [20], immunization against HPV was supposed to enter the Polish mandatory vaccination calendar in 2021, but the timing of the pandemic slowed the process, and it was not until 2023 that universal vaccination for adolescents of 12–13 years of both sexes comes into effect. The other forms of prevention, such as limiting active and passive smoking or periodic examinations in women, are also included in national preventive programs [17,18].

**Figure 1:**
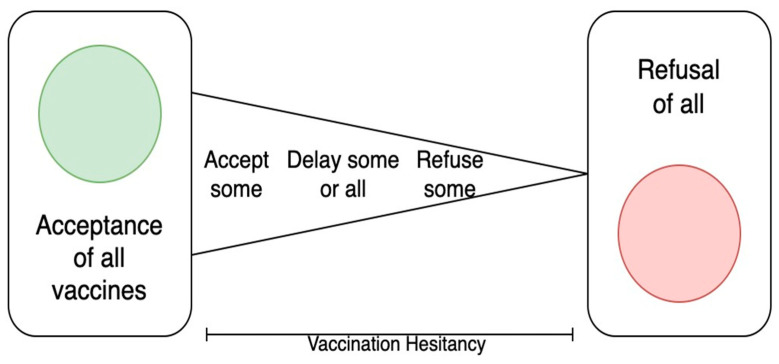
The continuum of vaccine hesitancy between full acceptance and outright refusal of all vaccines (based on SAGE WHO Report [23]).

One of the biggest threats to the effectiveness of immunization programs is the decline in the number of people vaccinated. The phenomenon of so-called vaccination hesitancy as defined by the WHO is the delay in the acceptance or refusal of vaccines despite the availability of vaccination services [23]. Vaccine hesitancy can vary in severity, as shown in the diagram in Figure 1.

**Figure 2:**
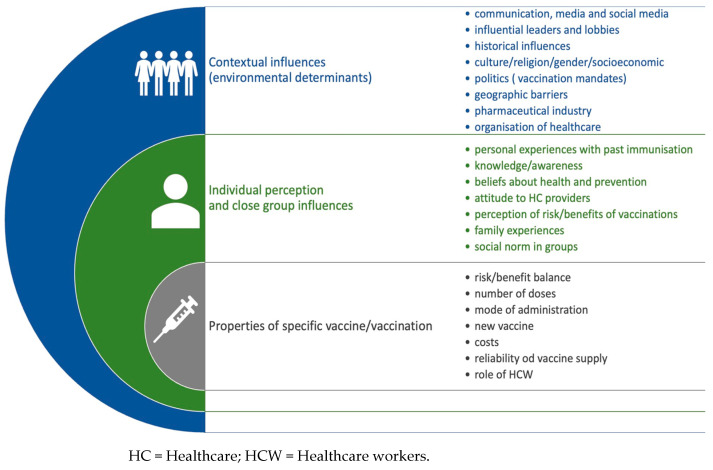
Determinants of vaccination hesitancy (based on WHO SAGE Report).

The problem is global, although the scale depends on the specific community. The reasons for the growing trends of refusing or delaying mandatory vaccinations in children are varied. The WHO Strategic Advisory Group of Experts on Immunization (SAGE) working group on vaccine hesitancy identified the main determinants affecting parents’ decisions to immunize their children [23]. They were divided into: those arising from the distant environment of the decisional parent, those related to personal perception or the views of the group with which they identify, and reasons associated with the specific characteristics of the vaccine itself (Figure 2).

### 1.1. A Group of Contextual Factors

They are resulting from the farther environment in a certain society. They are primarily related to the influence of the media and the ideas presented in the information or disinformation campaigns, including the significant contribution of social media. The message coming from opinion leaders, politicians, celebrities, scientists in various fields is of particular importance. Determinants of the decision to vaccinate can include historical, religious, cultural, gender, geographic, and socioeconomic factors—depending on the circumstances of the particular society. There are a number of other factors including confidence in the reliability of government institutions, trust in the intentions of pharmaceutical companies, the organization of preventive immunizations, and the assessment of the connection between the ruling authorities and the pharmaceutical industry.

### 1.2. A Group of Factors Connected with Individual Perception

The determinants of the closer surroundings, which are related to individual attitudes towards immunization of persons and the groups in which they function.

The level of parents’ knowledge and their awareness of the idea of how vaccination works, their familiarity with vaccine-preventable diseases (VPDs), and the potential side effects of vaccines are the factors that affect the specific individual balance of benefits and risks. The experiences of previous vaccinations in a given child or in other children in a close circle of family and friends are of great importance. All these factors influence the personal perception of whether vaccination is a safe and necessary procedure or not.

A significant effect is also exerted by the caregivers’ general attitude toward prophylaxis and health-promoting behavior, and the belief that some of these behaviors (e.g., breastfeeding, specific diet, vitamin supplementation, etc.) can be as effective as immunization.

### 1.3. A Group of Factors Directly Related to Specific Vaccines

Some vaccines are considered by communities to be more dangerous, ineffective, or unnecessary than others. In such situations, caregivers follow the vaccination program in the child/children, while refusal is associated with a specific vaccination. The individual perception of a particular vaccine as less safe than the others or redundant may depend on parents’ negative experiences with the product or their assessment of the disease threat as irrelevant. The conception that a vaccine, especially a new one, is untested, experimental and/or associated with the possibility of unknown adverse vaccine reactions, will increase the risk of wider refusal. An important determinant is the attitude of healthcare professionals: the way they provide information, and encourage or express doubts about the safety or potential benefit to the patient affect the final decision to vaccinate.

The WHO SAGE Working Group’s identification of the reasons influencing immunization decisions helps to develop programs to popularize knowledge about vaccine prevention and develop arguments and solutions that will reduce the phenomenon of vaccine hesitancy. According to experts, programs to counteract the phenomenon of vaccine abstention should be preceded by analyses and “tailored” to the needs of a specific community, taking into consideration environmental peculiarities that affect immunization decisions [23].

The study presented here was designed to examine parents’ attitudes toward HPV vaccination in the context of their decisions and beliefs about other childhood immunizations. An additional objective was to isolate the main beliefs and/or concerns in the parent community that determine vaccine hesitancy, especially towards HPV.

## 2. Materials and Methods

This study was conducted in a community of parents in Central Europe (Poland) in Gdynia, a city of 240,000 people. The municipal authorities had funded and promoted an HPV vaccination program for 13-year-old girls living in Gdynia since 2008, but not all parents took advantage of this opportunity. The author’s survey questionnaire was distributed between February 2023 and May 2023, on social media, on pages dedicated to the city’s various districts, on the pages of the Gdynia Health Center and in some Gdynia schools. The criterion for including respondents in the survey was the age of their children.

The survey targeted parents of children under 25 years of age covered by the city’s information campaign, about human papilloma virus and HPV vaccination, offered free of charge for girls. The content of the questionnaire referred to the parents’ vaccination decisions regarding mandatory and recommended vaccinations, including HPV and SARS-CoV-2.

In the first part, the parents answered questions related to with which vaccines and when their children were vaccinated. They expressed also opinions about the impact the COVID-19 pandemic on general attitudes toward immunization and the overall realization of the mandatory vaccination program among children.

In the second part, respondents determined their attitudes to common beliefs, regarding immunization, marking the extent to which they agreed with the mentioned statement on a 6-point scale. The last part was related to general data about the respondents—i.e., their gender, age, education (Supplementary Materials—questionnaire of the survey).

In the statistical analysis, the chi-square test of independence was used to check the relationship between qualitative characteristics. In the case of multiple groups, the non-parametric Kruskal–Wallis rank ANOVA test with post hoc test of multiple comparisons of mean ranks for all samples was used to compare the ordinal variables. Univariate logistic regression analyses were performed, and multivariate models were created using backward stepwise regression, for which AUCs were calculated. The first model described the effect of vaccination statements on a parent’s decision to vaccinate children with mandatory vaccinations. For this purpose, a binary variable was created based on the parent’s responses: “vaccinates children well”; “delays vaccination of their own accord or does not vaccinate”.

The second model described the impact of vaccination statements on a parent’s decision to vaccinate children against HPV. For this purpose, a binary variable was created based on the parent’s responses: “vaccinates children or declares such an intention”; “does not vaccinate”. In both models, the following were analyzed as adjusting variables: gender of the parent, age of the parent, education of the parent, and medical education. Statistically significant results were considered at *p* < 0.05. Statistica 13.3, STATA/SE 17 and MS Excel packages were used in the calculations.

## 3. Results

### 3.1. General Characteristics of the Study Group

A total of 250 completed questionnaires were obtained. Women were more likely to respond to the questionnaire, the majority of respondents marked higher education, and only 7% (*n* = 18) of people had a medical profession. The characteristics of the respondents are shown in Table 1.

### 3.2. Attitude of Surveyed Parents toward Mandatory Vaccinations

Respondents commented on the vaccination of their children, including the number of vaccinations and the possibility of a different implementation of vaccinations in subsequent children. Information was obtained on how and whether 425 children aged 0–25 were vaccinated. The results on the vaccination of children were calculated in relation to the number of children, while the results on the beliefs of the parents surveyed were calculated in relation to the number of respondents.

The majority of those who took part in the survey vaccinated their children with mandatory vaccinations from the vaccination calendar on time (*n*= 339). Only four children were not vaccinated with any vaccinations. Some of the respondents chose to skip some vaccination(s) or significantly delayed mandatory immunization: in 4.45% (*n*= 18), the delays were due to medical reasons, while for 11.8% (*n*= 57), delays were due to parental decisions—which meets the criterion of vaccine hesitancy.

Most often, parents decided to purchase acellular combination vaccines of 5in1 or 6in1 type (DTaP), replacing free vaccines with: DTP (Diphteria, Tetanus, Pertussis), Poliomyelitis, Hemophilus influenzae and Hepatitis B in separate injections. They also vaccinated their children with some or all of the recommended but not reimbursed vaccines: 44% of children *(n* = 172) received both alternative combination vaccines (5in1 or 6in1) and all of the additional recommended vaccines. Among the children, 16% *(n*= 63) did not receive any alternative or optional vaccinations at parental expense.

### 3.3. Beliefs about Immunization

In the questionnaire, respondents rated their attitudes toward 15 statements about immunization on a six-point scale: (1) disagree; (2) rather disagree; (3) somewhat disagree; (4) agree more than disagree; (5) rather agree; (6) fully agree. The charts below were created by taking opinions 1, 2, and 3 as opposing to 4, 5, and as coinciding with respondents’ beliefs. Responses indicating a pro-vaccination attitude were marked in green and those favoring abstention from vaccination were marked in red.

**Figure 3:**
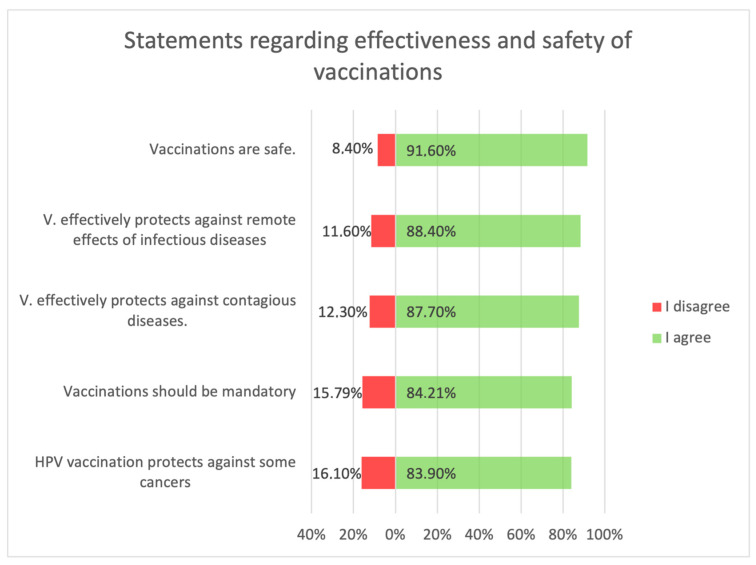
Statements regarding the safety and effectiveness of vaccinations.

**Figure 4:**
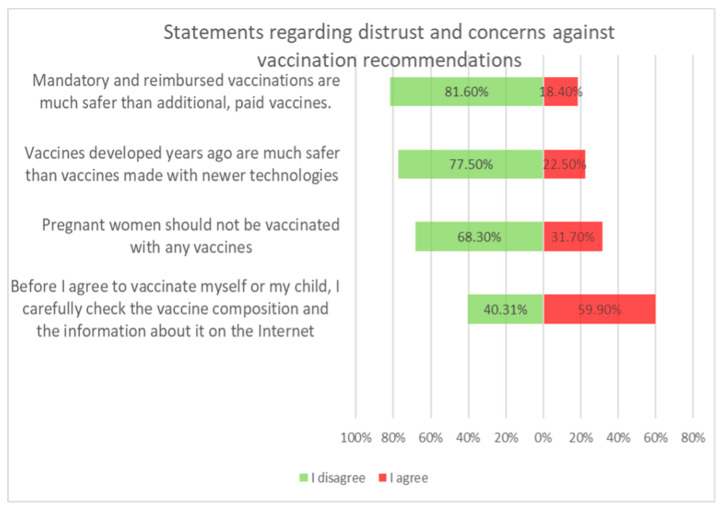
Statements regarding concerns against vaccinations.

**Figure 5:**
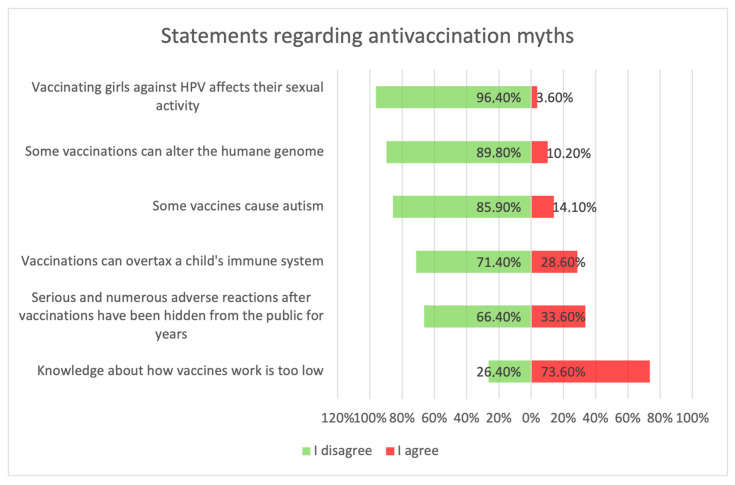
Statements about anti-vaccination myths.

Statements about vaccination were divided into opinions related to the safety and efficacy of vaccination (Figure 3), opinions related to distrustful attitudes toward vaccination and changes in official immunization recommendations (Figure 4), and untrue statements spread by anti-vaccination groups (Figure 5). The majority of respondents believed that immunization was safe, with only 8.4% (*n* = 21) holding the opposite view. Respondents generally had confidence in the idea of vaccinations and the effectiveness of immunizations: they believed that vaccinations are very effective in protecting against infectious diseases and their distant effects. The prevailing view among the surveyed parents was that vaccinations should be mandatory (Figure 3).

Vaccination and some recommendations regarding it, introduced in recent years (e.g., introduction of vaccination to pregnant women, introduction of new vaccines as mandatory, etc.), caused uncertainty or distrust among surveyed parents. Paid and additional vaccines were perceived by respondents to be safer than mandatory ones offered by the state. One-fifth of people (*n* = 47) held the opposite view. The view that vaccines developed many years ago are safer than those produced according to new technologies was shared by more than 22% (*n* = 56) of respondents. One-third (*n* = 79) believed that pregnant women should not be immunized. Nearly 74% thought that there was too little knowledge about how vaccines work, and tried to obtain information about planned vaccination on their own—from a leaflet or on the Internet (Figure 4).

Figure 5 shows parents’ attitudes toward false ideas that are spread by vaccine opponents (so-called “anti-vaccination myths”). For the most part, these are familiar to parents, and some of those surveyed agreed with these statements. Concerns about vaccine-induced autism were held by 12.4% (*n* = 31) of respondents, and about the possibility of altering the human genome by 10.2% (*n* = 28). Among the respondents, 28.6% *(n* = 63) were concerned that vaccinations could “overload children’s immune systems.” One of the more convincing statements introducing fears of vaccinations was the sentence that “Serious and numerous adverse reactions from vaccinations have been hidden from the public for years.” Among the parents, 33.6% (*n* = 84) agreed with this statement, including 12.8% *(n* = 32) with full conviction (response: “fully agree”).

### 3.4. Parents’ Knowledge and Attitudes vs. Timeliness of Mandatory Vaccinations

The influence of popular statements about immunization on a parent’s decision regarding mandatory immunization was analyzed using logistic regression. The parent’s decision took two states: “vaccinates their children on a calendar basis”; and “delays vaccination of their own accord or does not vaccinate”.

Univariate models showed that agreeing with statements I, II, X, XII, XIV had a positive effect on the chance of “timely vaccination”, while agreeing with statements VI, VII, VIII, IX, XI, XV had a negative effect on the correct vaccination of children (Table 2 and Table 3).

The interpretation of the multivariate model allowed us to conclude that, with an increase in the level of agreement with statement I “Immunization is safe”, the chance of timely vaccination of a child increases 1.67 times, and an increase in the level of agreement with statement XIV “Vaccination effectively protects against distant effects on infectious diseases” increases the chance of vaccination by 1.56 times, as recommended. The area under ROC curve for the model is 0.7756 (Table 3).

### 3.5. Parents’ Knowledge and Attitudes vs. HPV Vaccination of Children Compared to Mandatory Vaccines

HPV vaccination was performed less frequently than obligatory immunization. Parents of 20% of children (*n* = 78) indicated that they did not know about the possibility of vaccination. Among the children, 13% (*n* = 53) were vaccinated as part of the local government’s vaccination program, and 36% (*n* = 142) had not yet reached the age recommended for vaccination, but parents said they planned to administer HPV vaccines.

Respondents mostly knew that HPV vaccination protects against certain cancers: 59% (*n* = 147) fully agreed with this information, 16% (*n* = 40) pointed out that they disagreed with this statement. The anti-vaccine myth that the HPV vaccine affects girls’ sexual activity was not widespread—only 3.6% (*n* = 9) marked that they agreed, to varying degrees, with this statement.

The analysis of the association between mandatory immunization and HPV vaccination showed that among parents who “timely vaccinate their children with mandatory vaccination”, 51% *(n* = 100) have or will have their children vaccinated against HPV. Among parents who “do not vaccinate their children on time or at all”, only 37% (*n* = 14) have provided or declared to provide their children with additional protection against HPV. This relationship is not statistically significant (*p* = 0.103), perhaps due to the small number of people (*n* = 38) vaccinating in an untimely manner.

Another univariate logistic regression model showed that caregiver agreement with statements I, II, X, XII, XIV had a positive effect on the decision to vaccinate a child against HPV. The parent’s convergence with statements VI, VII, VIII, IX, XI had a negative effect on the decision to immunize against HPV (Table 4).

Table 5 shows a multivariate logistic regression model examining the impact of statements with the greatest influence on the decision to vaccinate against HPV. If the level of agreement with the opinion “HPV vaccination affects sexual activity” increases, the chance of “HPV vaccination” decreases by 2.07 times (=1/0.48). An increase in the level of agreement with the statement “HPV vaccination protects against cancer” increases the chance of “HPV vaccination” 1.26 times. Also, if the age of the parent increases, then the chance of “HPV vaccination” decrease 1.58 times (=1/0.63). The area under ROC curve for the model is 0.6979 (Table 5).

### 3.6. Attitudes toward Vaccinating Oneself against HPV

The fact that adults can also be vaccinated against the human papilloma virus was unknown to the majority of 60% (*n* = 144) of respondents. This information was known to 13 out of 18 respondents with medical education. Among the respondents, 9.1% (*n* = 22) are vaccinated against HPV, 56.8% (*n* = 137) indicated that they are considering the HPV vaccine, and 34% (*n* = 82) of respondents do not intend to receive it.

**Figure 6:**
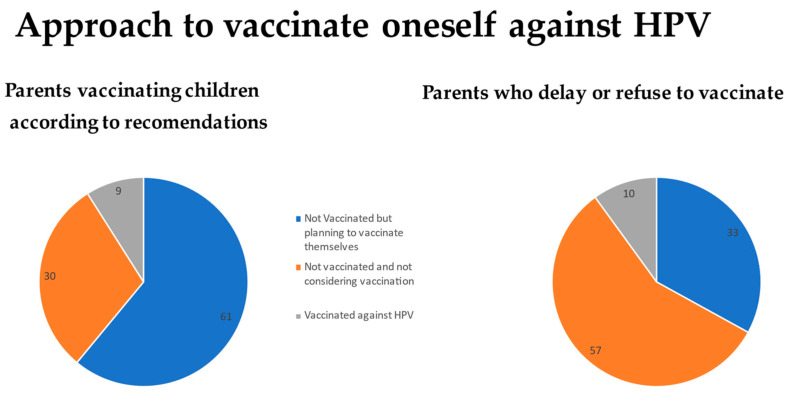
Approach of parents to vaccinate themselves against HPV vs. their attitude towards obligatory vaccinations for their children.

A correlation was shown between parents’ attitudes regarding the timeliness of their children’s mandatory vaccinations and attitudes toward vaccinating themselves against HPV. Statistical significance was shown between “timely” and “not timely mandatory vaccination of children” and parental vaccination against HPV (*p* = 0.004). Among parents who “timely vaccinate their children”, 9% (*n* = 18) were vaccinated against HPV, 61% (*n* = 121) were considering it, and 30% (*n* = 59) did not plan to vaccinate themselves against HPV. Among parents who “do not vaccinate their children on time or at all”, 10% (*n* = 4) were vaccinated against HPV, 33% (*n* = 13) were considering doing so, and 56% (*n* = 22) did not plan to (Figure 6).

The results of the analysis of the relationship between respondents’ attitudes towards the 15 vaccination statements and attitudes about vaccinating themselves against HPV are presented in Table 6.

### 3.7. Survey Respondents’ Approach to Vaccination against COVID 19

Among the respondents, 85.2% (*n* = 207) had been vaccinated against COVID-19. The question about vaccinating children against SARS-CoV-2 indicated that it was about underage children, where the vaccination status was due to a parent’s decision. Only 54.5% (*n* = 128) of parents decided to vaccinate their children against COVID-19 and 9.8% (*n* = 23) declared that they would vaccinate them. It was shown that, between persons not vaccinated against HPV, but considering the possibility, and those who are not vaccinated and do not plan to be immunized, there are statistically significant differences in 11 opinions about vaccinations out of 15: I, II, VI, VII, VIII, IX, X, XI, XII, XIV, XV.

In contrast, there is a difference only in two opinions between those who have been vaccinated against HPV and those that are considering it: VI—“Knowledge about how vaccines work is too low” and XII—“Vaccination is very effective in protecting against infectious diseases “ (Table 6). Moreover, 35.7% (*n* = 84) indicated that their children were not vaccinated against COVID-19. Among those who had vaccinated their children against HPV, 70% (*n* = 78) had vaccinated against COVID-19 or declared that they would. Among those who had not vaccinated their children against HPV, this percentage was lower at 58% *(n* = 67). This relationship was close to statistical significance (*p* = 0.062).

## 4. Discussion

Respondents’ knowledge of HPV vaccination was quite high compared to the results obtained in a 2022 survey of parents of Polish teenagers conducted by the Ministry of Health [25,26]. Only 37.5% of respondents from the Polish nationwide survey knew how to specify the diseases that HPV causes, while 83.9% of parents from Gdynia knew that HPV vaccination protects against certain cancers. These results are probably due to the fact that free HPV vaccinations were carried out in the city of Gdynia since 2008, and there is an ongoing information campaign about it.

In other countries, the awareness of HPV as the reason for oncogenesis is greater, but also not satisfying: for instance, in Greece, 50% respondents knew the relation [27]; in Northern Cyprus, 43% knew the relation [28].

Among the respondents of our study, 20% did not receive the information about the possibility to immunize children against HPV. The option to be vaccinated against the papilloma virus in adulthood was new to most of the respondents. A significant number of parents, unvaccinated against HPV, began to consider such a vaccination for themselves. The dissemination of information about possible HPV vaccinations for adult people is extremely important. The risk of infection with an oncogenic virus is most often associated with a youthful period of increased sexual activity but in fact it can occur at any stage of one’s life. More importantly, the impact of HPV vaccination on ongoing infection has been proven. The immunization supports the elimination of oncogenic viruses [24,29].

The guardians who are considering the possibility of vaccinating themselves in comparison to the respondents who do not intend to get vaccinated against HPV had divergent views towards most of the statements about immunization included in the questionnaire.

The confidence in the effectiveness of immunization, and therefore the possibility of preventing infections and their distant complications, including cancer, is conducive to the correct, timely vaccination of children, and it is worth increasing information on this topic [25,26]. In increasing the acceptance of HPV vaccination, the most helpful in providing comprehensive information seems to be information campaigns aimed at parents of children, young adults, and medical personnel [30,31,32]. In some countries—such as Northern Ireland [33]—the immunization nurses at schools are delivering education about vaccinations and about HPV- related diseases. In Poland, there is currently a discussion on forms of educating young people about the topics related to sex life, including sexually transmitted diseases and their prevention.

The individual decision to vaccinate oneself or one’s child depends on the peculiar balance of benefits and risks associated with vaccination. Respondents who perceived vaccination as safe and considered it effective in preventing disease and its remote effects were more likely to make a pro-vaccination choice. Those who were not convinced of the need or effectiveness of vaccines and feared side effects perceived vaccination as a procedure with significant risks versus questionable benefits [34].

Vaccine-preventable diseases (VPDs) are mostly unknown to modern parents because they are extremely rare thanks to widespread immunization. The success of immunization programs paradoxically leads to questioning the need for vaccination [19,34].

Concerns about vaccinations are in line with the beliefs of some respondents, who do not trust full, reliable knowledge about vaccinations or official statistics regarding the number of adverse vaccine reactions. Suspicion of the intentions of pharmaceutical companies and the honesty of institutions reduces the motivation to vaccinate according to recommendations [35,36]. Confidence in the safety of vaccination is one of the most important factors influencing the decision to vaccinate a child. Fear of the danger of complications fosters attitudes of withholding vaccination, including HPV vaccination [35,37,38]. The parental fear of side effects is also the most important factor inhibiting teen vaccinations against HPV in other countries—such as Romania [39], Japan [40], or Sweden [41].

Anti-vaccine myths are often linked to a particular vaccine and its characteristics, leading to the perception that it is extremely risky. The myth of autism as a consequence of vaccination first appeared as a result of published by A. Wakefield studies on the MMRII vaccine. Research results were later found to be falsified. A number of later studies demonstrated the lack of a link between MMR vaccination and autism diagnosis [42,43,44,45] and A. Wakefield admitted to manipulation and fraud. However, this myth continues to be promoted by the anti-vaccine lobby and was initially linked to vaccination against measles, mumps, rubella and—in later years—also to vaccination containing thimerosal. A small number of respondents to the presented survey identified with this belief.

The COVID-19 vaccination was produced at a rapid pace, using m-RNA technology, unknown in non-medical circles. The concerns appeared that the vaccine was not adequately tested and that the use of nucleic acids could introduce changes to the human genome [45]. Doubts were mainly spread on the Internet. The flood of misinformation on social media was referred to as an infodemic. Some of the posts appeared online as a result of bots created by the anti- or pro-vaccine lobby [46,47].

Vaccination for the human papilloma virus is associated with the myth of the earlier sexual initiation of the vaccinated. HPV is a sexually transmitted virus (STI), and concerns have been raised that vaccinated girls may perceive HPV immunization with parental consent as an acceptance of initiation at a young age and/or feel encouraged to begin their sexual lives early and engage in risky behaviors—such as not using condoms or frequently changing sexual partners [48,49,50,51,52]. Studies on this topic have found no association of HPV vaccination with earlier sexual activity [49,50,51,52,53]. Some studies have reported results indicating that vaccinated girls began sexual activity later than unvaccinated girls and had a lower tendency to engage in risky behavior, which has been linked no to the vaccines effects but to environmental factors, such as awareness and the socioeconomical status of their families [51,52].

A myth related to “immune system overload” has been linked to the concern that children receive too many vaccines and this may weaken their immunity. No such connection has been documented [4]. Vaccines contain either proteins or de-virulent viruses that are unable to weaken the immunity of the vaccinated person, but help produce antibodies to specific microorganisms. The solution seemed to be combining vaccines with each other in combination preparations, which reduces the number of injections and increases parental acceptance. Studies have also been performed, confirming the safety of the co-administration of multiple vaccines during a single visit [53,54]. Often, however, anxious parents, in order not to expose their child to too much—in their opinion—to an overstimulation of the immune system, decide to separate vaccines, one for each visit. This procedure neither reduces the risk of adverse reactions nor improves the produced immunity, but increases the number of visits to the vaccination center and prolongs the time for the child to achieve full immunity, and is a classic example of vaccine withholding [52,53,54,55,56,57,58].

A parent’s compliance with anti-vaccine myths decreases the chance of vaccinating their child, especially on the recommended schedule [56].

The group of parents vaccinated against HPV differed from those not planning to vaccinate against HPV significantly less than those considering vaccinating themselves. Parents already protected against HPV vaccinated their children on time as well as delayed immunization to a similar extent as the reluctant group. HPV vaccination status did not increase these parents’ compliance with pro-vaccination statements. There are no data on what proportion of this group was vaccinated in their teenage years by their parents versus by their own decision. No studies have been conducted on the association between parents’ HPV vaccination status and their decisions to vaccinate their children or their attitudes toward immunization.

### Limitations

Limitations of our survey were the small percentage of respondents who did not vaccinate their children—making some of the results obtained not statistically significant.

Questions about decisions respondents had made in the past were subject to the risk of s associated with forgetting the details of their children’s vaccinations.

## 5. Conclusions

(1) Knowledge of the possibility of vaccinating adults against HPV was not widespread, including among respondents with a pro-vaccination attitude. Awareness campaigns should emphasize the possibility of HPV vaccination for adults as well, especially since they do not only have a preventive effect, but also have a potentially therapeutic role.
(2) Factors positively influencing vaccination against HPV were respondents’ agreement with statements emphasizing vaccine safety and vaccine efficacy, and younger parental age. Most important for the positive decision to vaccinate a child was the awareness that the HPV vaccine protects against cancer; information about the human papillomavirus infection and its carcinogenic effects should be more widely publicized, together with the safety data of HPV vaccines.
(3) Factors negatively influencing vaccination against HPV were parents’ agreement with statements presenting concerns about vaccination, anti-vaccine myths, and the parents’ older age. The most significant influence on the negative decision to immunize was the respondent’s compliance with the opinion that HPV vaccines can affect the child’s sexual activity.
(4) The timeliness of mandatory immunization in children was associated, as with HPV vaccination, with beliefs about the safety and efficacy of vaccination. Concerns or belief in anti-vaccine myths, especially related to beliefs about the side effects of vaccination, had a negative impact on the timely vaccination of children according to the vaccination schedule. The greater availability of information on adverse vaccine reactions—both reported by medical professionals and patients themselves—could increase confidence in the data on this topic. Fear of vaccine side effects is one of the most significant determinants holding parents back from immunization, including against HPV.
(5) It is important to increase the competence of medical personnel in communicating information about vaccination and its safety, as well as their ability to talk to fearful and/or skeptical parents. It should be emphasized, however, that the fight against vaccination misinformation must also take place on social media, with use of modern methods, as the Internet is one of the main sources of information for many parents.

## Figures and Tables

**Table 1:** Respondents’ characteristics.

Variable	
Female	217 (86.8%)
Age (years)	
18–25	7 (2.8%)
26–35	43 (17.1%)
36–45	124 (49.4%)
45–60	76 (30.3%)
Education	
Primary education	4 (1.6%)
Secondary education	37 (14.8%)
Higher education Bachelor’s degree	19 (7.6%)
Higher education level 2 or 3	190 (76%)
Number of children	
1	91 (37.8%)
2	117 (48.1%)
3	28 (11.6%)
4	4 (1.7%)

**Table 2:** Univariate logistic regression model examining compliance with vaccination declarations in the context of attitudes toward mandatory vaccination.

Statements *	OR	95% CI	*p*-Value
I	1.98	1.48–2.65	>0.001 **
II	1.60	1.31–1.96	>0.001 **
III	1.10	0.86–1.41	0.433
IV	0.89	0.71–1.12	0.329
V	0.90	0.73–1.10	0.310
VI	0.72	0.59–0.87	0.001 **
VII	0.73	0.60–0.89	0.002 **
VIII	0.73	0.57–0.92	0.007 **
IX	0.71	0.53–0.96	0.024 **
X	1.35	1.08–1.68	0.008 **
XI	0.65	0.52–0.81	>0.001 **
XII	1.42	1.14–1.77	0.002 **
XIII	0.96	0.78–1.19	0.709
XIV	1.84	1.41–2.41	>0.001 **
XV	0.55	0.43–0.72	>0.001 **
Sex (male)	3.01	0.69–13.20	0.144
Parents’ age	1.22	0.75–1.98	0.422
Education	1.43	0.97–2.10	0.069
Medical education	0.47	0.16–1.41	0.180

* I—Immunizations are safe; II—Vaccinations should be mandatory; III—Mandatory and reimbursed vaccinations are much safer than additional, paid vaccines; IV—Vaccines developed years ago are much safer than vaccines made with newer technologies; V—Serious and numerous adverse reactions after vaccinations have been hidden from the public for years; VI—Knowledge about how vaccines work is too low; VII—Pregnant women should not be vaccinated with any vaccines; VIII—Some vaccinations can alter the human genome; IX—Vaccinating girls against HPV affects their sexual activity; X—HPV vaccination protects against some cancers; XI—Some vaccines cause autism; XII—Vaccination is very effective in protecting against infectious diseases; XIII—Before I agree to vaccinate myself or my child, I carefully check the composition of the vaccine and the information about it on the Internet; XIV—Vaccination effectively protects against the remote effects of infectious diseases; XV—Vaccinations can overtax a child’s immune system. ** The differences are statistically significant at *p* < 0.05. OR, odds ratio; CI, confidence interval.

**Table 3:** Multivariate logistic regression model examining the effect of respondents’ compliance with positive vaccination statements on the timely completion of mandatory vaccinations.

Statements *	OR	95% CI	*p*-Value
I	1.67	1.22–2.28	0.001 **
XIV	1.56	1.15–2.11	0.004 **

* I—Immunizations are safe; XIV—Vaccination effectively protects against the remote effects of infectious diseases; ** The differences are statistically significant at *p* < 0.05. OR, odds ratio; CI, confidence.

**Table 4:** Univariate logistic regression model examining attitudes toward vaccination statements in the context of attitudes towards the HPV vaccination of children.

Statements *	OR	95% CI	*p*-Value
I	1.33	1.04–1.71	0.024 **
II	1.37	1.14–1.66	0.001 **
III	0.95	0.80–1.13	0.577
IV	0.83	0.70–0.99	0.039 **
V	0.99	0.85–1.14	0.847
VI	0.78	0.67–0.91	0.002 **
VII	0.81	0.69–0.95	0.009 **
VIII	0.70	0.56–0.88	0.002
IX	0.45	0.30–0.67	>0.001 **
X	1.39	1.13–1.70	0.002 **
XI	0.72	0.59–0.89	0.002 **
XII	1.26	1.03–1.54	0.022 **
XIII	0.94	0.80–1.10	0.424
XIV	1.28	1.03–1.60	0.027 **
XV	0.89	0.74–1.07	0.213

* I—Immunizations are safe; II—Vaccinations should be mandatory; III—Mandatory and reimbursed vaccinations are much safer than additional, paid vaccines; IV—Vaccines developed years ago are much safer than vaccines made with newer technologies; V—Serious and numerous adverse reactions after vaccinations have been hidden from the public for years; VI—Knowledge about how vaccines work is too low; VII—Pregnant women should not be vaccinated with any vaccines; VIII—Some vaccinations can alter the human genome; IX—Vaccinating girls against HPV affects their sexual activity; X—HPV vaccination protects against some cancers; XI—Some vaccines cause autism; XII—Vaccination is very effective in protecting against infectious diseases; XIII—Before I agree to vaccinate myself or my child, I carefully check the composition of the vaccine and the information about it on the Internet; XIV—Vaccination effectively protects against the remote effects of infectious diseases; XV—Vaccinations can overtax a child’s immune system. ** The differences are statistically significant at *p* < 0.05. OR, odds ratio; CI, confidence interval.

**Table 5:** Multivariate logistic regression model examining the influence of the statements having the greatest impact on the HPV vaccination decision.

Statements *	OR	95% CI	*p*-Value
IX	0.48	0.32–0.73	0.001 **
X	1.26	1.02–1.57	0.031 **
Parents’ age	0.63	0.42–0.95	0.028 **

* IX—Vaccinating girls against HPV affects their sexual activity; X—HPV vaccination protects against some cancers; ** The differences are statistically significant at *p* < 0.05. OR, odds ratio; CI, confidence interval.

**Table 6:** The compliance of respondents with the statements about vaccinations vs. the attitude to vaccinate themselves against HPV.

Statement	1. I am Vaccinated against HPV 2. No But I Consider to Vaccinate 3. No and I Do Not Plan to Vaccinate	*p*-Value 1 vs. 2 1 vs. 3 2 vs. 3
I—Immunizations are safe	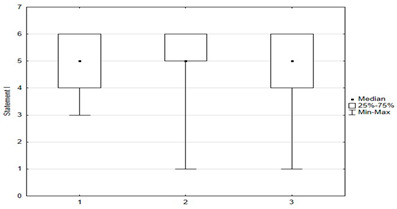	2 vs. 3 *p* = 0.004
II—Vaccinations should be mandatory	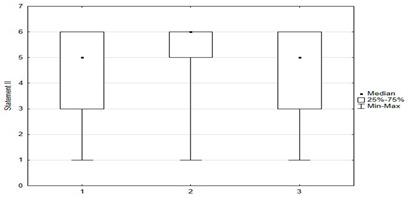	2 vs. 3 *p* < 0.001
VI—Knowledge about how vaccines work is too low	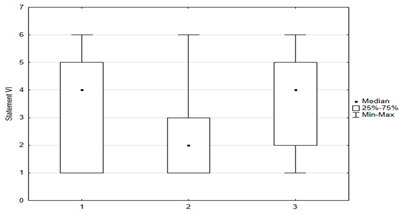	1 vs. 2 *p* = 0.014 2 vs. 3 *p* < 0.001
VII—Pregnant women should not be vaccinated with any vaccines	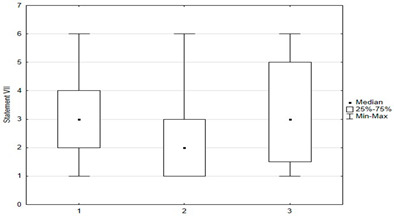	2 vs. 3 *p* = 0.001
VIII—Some vaccinations can alter the human genome	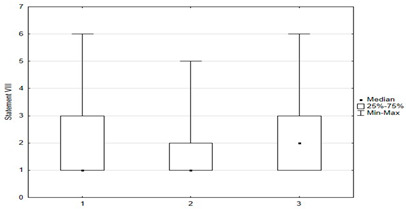	2 vs. 3 *p* = 0.021
IX—Vaccinating girls against HPV affects their sexual activity	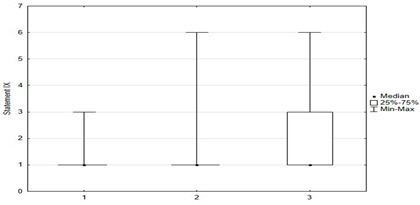	2 vs. 3 *p* = 0.010
X—HPV vaccination protects against some cancers	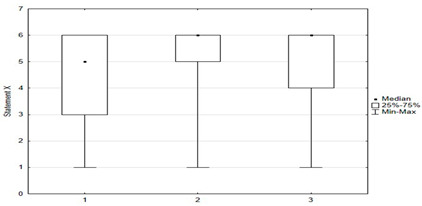	2 vs. 3 *p* = 0.029
XI—Some vaccines cause autism	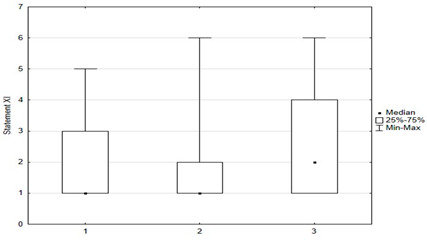	2 vs. 3 *p* = 0.004
XII—Vaccination is very effective in protecting against infectious diseases	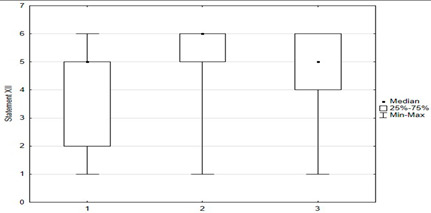	1 vs. 2 *p* = 0.004 2 vs. 3 *p*=0.022
XIV—Vaccination effectively protects against remote effects of infectious diseases	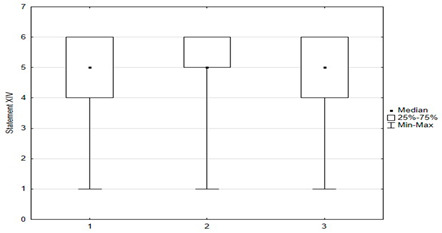	2 vs. 3 *p* = 0.005
XV—Vaccinations can overtax a child’s immune system	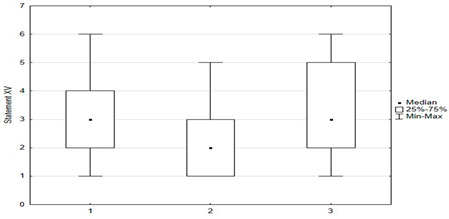	2 vs. 3 *p* = 0.023

## Data Availability

Not applicable.

## Supplementary Materials

The following supporting information can be downloaded at: https://www.mdpi.com/article/10.3390/medicina59101755/s1.
