# Supplementary material for: Analysis of Parents’ Attitudes and Knowledge toward Immunization and How These Factors Influence Their Decisions to Vaccinate Their Children against Human Papilloma Virus (HPV)
Source: Medicina (Kaunas). 2023 Sep 30;59(10):1755. doi: 10.3390/medicina59101755 (PMC10608555; doi:10.3390/medicina59101755)
Supplement: Supplementary file 1 [file medicina-59-01755-s001.zip › medicina-2593654-Supplementary Materials.pdf]

Supplementary Materials

Opinions of parents from Gdynia about preventive vaccinations, including: vaccination against human papillomavirus (HPV)

The program for financing preventive vaccination against HPV (Human Papilloma Virus = human papillomavirus) has been implemented for years for 13 and 14-year-old girls in Gdynia. This virus is responsible for causing cervical cancer in women, as well as other cancer of the genital area and the head and neck in both sexes.

The survey is targeted at residents of Gdynia, who are parents of children between 0 and 25 years old, and the time to spend participating in the study is about 6-8 minutes. Every opinion is important to us; Your answers to questions will remain anonymous (results will only be presented in the form of aggregate statistical summaries).

Warning! Choose the right answer for each question by selecting the appropriate circle or entering the appropriate number.

1. Are you the parent of a child / children up to 25 years old?

- Yes
- No

2. Please provide the number of your children: …

3. Please provide the sex of each child :

- Child 1 - Male / Female
- Chlid 2 – Male /Female
- ….

4. Please indicate the appropriate age for each child:

	0-5 yrs old	6-10 yrs	11-15yrs	15-18yrs	18-25 yrs old
Child 1					
Child 2					
…					

Mandatory vaccinations for children are free and they are defined in the Protective Vaccination Program as the so-called vaccination calendar. At the same time, the above document lists vaccines that are available and recommended for administration to children of the right age, but are not refunded - i.e. they can be bought by parents or are sometimes available, e.g. as part of local preventive programs.

5. Have your child / children been vaccinated with compulsory protective vaccinations from the vaccination calendar? (please indicate only one answer option for each child) :

Child1:

- no, my child has not been vaccinated with any protective vaccinations;
- yes, my child was vaccinated, but not all vaccines out of those intended for his/her age;

- yes, but the child was vaccinated with long delays and / or not all vaccines intended for its age - for medical reasons;
- yes, but it is vaccinated with long delays and / or not all vaccines intended for its age - for mainly NON-medical reasons;
- yes, but it is vaccinated on dates chosen by parents, much later than recommended;
- yes, the child is vaccinated and generally on time.

Child 2:…(the same options to eventual following children)

6. Was your child /children vaccinated with non-refundable vaccines - at the expense of the parents? (please indicate only one answer option for each child):

Child 1:

- no
- yes, with paid equivalents of mandatory vaccines (e.g. 5in1 or 6in1 vaccines);
- yes, with some recommended, additional vaccinations
- yes, with both paid equivalents of mandatory vaccination and some selected, recommended, vaccines

Child2: …(the same options to eventual following children)

7. Has your child / children been additionally vaccinated against HPV? (please indicate only one answer option for each child):

Child 1:

- has not been vaccinated and I do not plan such vaccination at all;
- no, because the vaccination was only refunded form girls;
- no, because I knew nothing about such a possibility;
- no, but I plan to vaccinate the child against HPV in recommended age;
- yes, the child was vaccinated as part of a free local government program;
- yes, at the expense of parents;
- I do no know.

Child 2: …

8. Did you know that there is also the possibility of vaccination against HPV virus for an adult?

- NO
- YES

9. Have you decided to vaccinate yourself with the HPV vaccine for a fee?

- NO and I do not plan to get vaccinated against HPV.
- NO, but I am considering such a possibility.
- I am vaccinated against HPV.

10 and 11 XXX- irrelevant to this article.

12. Please specify your attitude to each of the following statements on a scale of 1-6, where:

1- I strongly disagree, 2- I rather disagree, 3 I disagree a little -, 4 – I agree more than I disagree, 5. I rather agree, 6- I fully agree:

I. Immunizations are safe;
II. Vaccinations should be mandatory;
III. Mandatory and reimbursed vaccinations are much safer than additional, paid vaccines;
IV. Vaccines developed years ago are much safer than vaccines made with newer technologies;
V. Serious and numerous adverse reactions after vaccinations have been hidden from the public for years;
VI. Knowledge about how vaccines work is too low;
VII. Pregnant women should not be vaccinated with any vaccines;
VIII. Some vaccinations can alter the human genome;
IX. Vaccinating girls against HPV affects their sexual activity;
X. HPV vaccination protects against some cancers;
XI. Some vaccines cause autism;
XII. Vaccination is very effective in protecting against infectious diseases;
XIII. Before I agree to vaccinate myself or my child, I carefully check the composition of the vaccine and the information about it on the Internet;
XIV. Vaccination effectively protects against the remote effects of infectious diseases;
XV. Vaccinations can overtax a child's immune system.

13. – XXX under analysis to next aricle

14. Please select the best 3 sources of information about vaccinations according to you:

- doctors and nurses of vaccination points;
- independent medics, not associated with vaccination process;
- vaccinated persons or parents of vaccinated children;
- people associated with alternative medicine and natural methods of increasing immunity;
- social media;
- TV programmes;
- the magazines for parents;
- websites of medical societies;
- leaflets and information about contents of vaccines;
- websites of the World Health Organization WHO.

Information about the person completing the questionnaire (select the correct answer):

15. Sex: Male/Female

16. Age: 18-25; 26-35; 36-45; 46-60; more than 60.

17. Level of education:

- primary education
- lower secondary education
- essential professional or industry education
- upper secondary/ upper secondary professional education
- bachelor's degree
- 2nd(MA) or 3rd (PhD) degree higher education.

18. Medical education: Yes/ No

Thank you for your time!
